# Age and sex differences in the association between access to medical care and health outcomes among older Chinese

**DOI:** 10.1186/s12913-018-3821-3

**Published:** 2018-12-29

**Authors:** Xufan Zhang, Matthew E. Dupre, Li Qiu, Wei Zhou, Yuan Zhao, Danan Gu

**Affiliations:** 1 Ginling Colleague, Nanjing Normal University, Nanjing, China; 2 Department of Population Health Sciences and Department of Sociology, Duke University, Durham, NC USA; 3 Independent Researcher, New York, NY USA; 4 School of Geographical Science Ginling College, Nanjing Normal University, and Jiangsu Center for Collaborative Innovation in Geographical Information Resource Development and Application Nanjing, Nanjing, China; 5 United Nations Population Division, Two UN Plaza, New York, NY DC2-1910 USA

**Keywords:** Access to healthcare, Disability, Mortality, Older adults, Oldest-old, Gender differences, Age differences, China, CLHLS

## Abstract

**Background:**

Whether the association between access to medical care and health outcomes differs by age and gender among older adults in China is unclear. We aimed to investigate the associations between self-reported inadequate access to care and multiple health outcomes among older men and women in mainland China.

**Methods:**

Based on four latest waves available so far from a national longitudinal study in mainland China in 2005–2014, we used multilevel random-effect logistic models to estimate the contemporaneous relationships between inadequate access to care and disabilities in instrumental activities of daily living (IADL) and cognitive impairment in men and women at ages 65–74, 75–84, 85–94, and 95+, separately. We also used multilevel hazard models to investigate the relationships between reported access to care and mortality in 2005–2014. Nested models were used to adjust for survey design, sociodemographic background, enrollment in health insurance, and health behaviors.

**Results:**

Approximately 6.5% of older adults in China reported inadequate access to care in the period of 2005–2014; and the percentages increased with age and were higher among women at older ages (≥75 years). Overall, older adults with self-reported inadequate access to care had greater odds of IADL and ADL disabilities and cognitive impairment than those with adequate access to healthcare. The elevated odds ratios (ORs) in men were higher in middle-old (75–84) and old-old (85–94) age groups compared to other age groups; whereas the elevated ORs in women were higher in young-old (65–74) and middle-old (75–84) age groups. The relationship between access to care and the health outcomes was generally weakest at the oldest-old ages (95+). Inadequate access to care was also linked with higher mortality risk, primarily in adults aged 75–84, and it was somewhat more pronounced in women than in men.

**Conclusions:**

Increased odds of physical disability and cognitive impairment and increased risk of mortality are linked with inadequate access to care. The associations were generally stronger in women than in men and varied across age groups. The findings of the present study have important implications for further improving access to health care and improving health outcomes of older adults in China.

## Background

Numerous studies from many populations have shown that adequate access to medical care promotes better health [1–8] and that inadequate access to medical care is linked with greater psychological distress [9, 10], readmission of hospitalization [11], lower levels of self-rated health and life satisfaction [12], worse physical health [3, 4, 13, 14], and overall higher rates of morbidity and mortality [6, 14–16]. Adequate access to medical care is particularly critical to health conditions and well-being of older adults who generally have greater needs for care and treatment to manage diseases than their younger counterparts. However, our understanding of how differences in access to medical care is associated with different health indicators at older ages is lacking and is largely based on studies from western countries.

Existing studies have shown that reported access to (or actual utilization of) healthcare is not uniform across subgroups in many populations—e.g., by sex, age group, race/ethnicity, and urban/rural residence [14, 17–29]. Accordingly, a great deal of research suggests that women are more likely to use physician visit services and home healthcare services than men; whereas men are more likely to rely on hospital care than women [2, 28, 30–34]. Likewise, research has shown that the factors influencing access to medical care vary by age in later life. For example, higher co-pay and lack of insurance coverage are often attributed to lower self-reported accessibility to healthcare among the youngest-old adults (aged 60–69); whereas greater needs due to poor health and lack of transportation are more often attributed to lower accessibility of care among the oldest-old adults (aged 80 or older) [35].

Despite ample literature demonstrating age and sex differences in healthcare access and utilization [2, 28, 30, 36–39], as well as age and sex differences in health status and mortality [2, 40–42], there is limited evidence of whether and to what extent the association between healthcare access/utilization and health varies by age or gender. Even less is known in developing countries with rapidly aging populations such as China. The unique healthcare system in China (see Appendix) provides an informative context to understand how population dynamics—such as age and sex—can influence the degree to which access to care has implications for subsequent health outcomes.

A growing body of research now focuses on self-reported access to health care to mitigate the counterintuitive findings that have been shown with the actual use of healthcare or medical care at older ages [7, 43]. Self-reported access to care reflects an individual’s context and perceptions about whether they could obtain needed healthcare services when needed. It captures information about (i) whether the use of healthcare meets their needs, (ii) whether they could get timely treatment, (iii) whether there are any barriers or delays in receiving care, (iv) whether the services they received are satisfactory, and (v) other perceived dimensions in accessing care that the actual use of healthcare does not capture [44–47]. Collectively, reported access to care is a multidimensional measure that provides information beyond utilization that better informs efforts to improve access to care and person-centered approaches to care.

A recent study in China showed that rural older adults were less likely to report adequate access to medical care than urban older adults, and that the negative consequences on health of inadequate access to medical care were more pronounced in rural older adults than in urban older adults [14]. The objective of the current study is to further examine whether reported (in)adequate access to medical care is associated with multiple health outcomes (i.e., physical and function, cognitive function, and mortality) by age and sex based on four latest waves available from a nationally representative survey focusing on older adults from mainland China. To our knowledge, our current study is the pioneer to examine age and sex differences in the relationship between reported (in)adequate access to care and multiple health outcomes among older Chinese. The results of this study have potentially important implications for developing interventions and/or policies to address age and sex disparities in access to care in China and reducing disability, cognitive impairment, and overall mortality at older ages.

## Methods

### Data

We used the latest four waves of data available so far from the Chinese Longitudinal Healthy Longevity Survey (CLHLS) in mainland China in 2005, 2008/2009, 2011/2012, and 2014. Because information on the respondents’ access to medical care was not measured consistently and the information on health insurance was not collected in the first three waves (1998, 2000, and 2002, we did not include them in the analysis. The CLHLS was conducted in a randomly selected half of the counties/cities in 22 of 31 provinces. Nine other provinces were excluded from the sampling design because of concerns about the accuracy of age reporting at the very oldest ages [37]. In the 2008 wave and later, an additional county (with 95% Han ethnicity) was included in the survey. The total population of these 23 provinces in the 2010 census accounted for nearly 90% of China’s total population.

The CLHLS was uniquely designed to oversample adults aged 80 and older to ensure a sufficient sample size for studying aging and longevity. In addition, the sample was replenished in each wave to replace participants who were deceased or lost to follow-up. The survey was conducted through door-to-door interviews by the CLHLS team—which consisted of a trained professional staff member and a local nurse/doctor (or medical student). To ensure the accuracy of age-reporting of a respondent, multiple sources (whenever available) were used for verification, including birth certificates, genealogical documents, and school enrollment archives. The overall response rate in each wave was consistently above 95%. Additional information about the detailed sampling design, survey procedures, and the overall data quality of the survey can be found elsewhere [37].

The final valid analytic sample consisted of 25,567 older adults who were interviewed in any wave from 2005 to 2014 and were aged 65 or older at the interview—contributing 46,549 total observations. The four waves of data were pooled together to improve the robustness of the age-sex-specific estimates. This included large subsamples of women at ages 65–74 (*n* = 2149), 75–84 (*n* = 2206), 85–94 (*n* = 4439) and 95+ (*n* = 6023) and men at ages 65–74 (*n* = 2463), 75–84 (*n* = 2409), 85–94 (*n* = 3910) and 95+ (*n* = 1968). Ethics approval was not needed for this study because it uses publicly available data.

### Measurements

#### Access to medical care

The United States National Academies of Sciences, Engineering, and Medicine [48] defines healthcare as the inclusion of a wide array of services—consisting of preventive care, mental health services, dental care, and other community services that promote health. Access to health or medical care in the CLHLS consisted of several questions: Whether they could get (in)adequate access to medical care when needed, the number of hospitalizations in the last 2 years, the type of insurances purchased, and so on. In the present study, we focused on the first question to measure whether the respondent reported adequate access to care (yes vs. no). To minimize missing data, the CLHLS used proxy responses (i.e., permitting next-of-kin or other family members, etc.) to gather data on access to care for the sampled older adults who were unable to provide this information due to sickness. The weighted proportion with proxy responses for this question was approximately 5% in the last four waves of the CLHLS. Preliminary analysis indicated a comparable result of this measure with another national survey.

#### Health outcomes

##### IADL, ADL, and cognitive impairments

We used a modified version of Lawton’s scale to measure impairments in instrumental activities of daily living (IADL) among Chinese older adults [37], which included eight following self-reported activities: (a) visiting and talking to neighbors, (b) shopping, (c) preparing meals, (d) doing laundry, (e) walking one kilometer, (f) lifting a 5-kg object, (g) crouching and standing up three times, and (h) using public transportation. The items had three response categories: “able to do without help,” “need some help,” and “need full help.” We classified a respondent as IADL disabled if he/she reported needing any help in any item (coded as 1), otherwise the respondent was considered as not IADL disabled (coded as 0). Impairment in activities of daily living (ADL) was measured using the Katz scale, which included the following six activities: (a) bathing or showering, (b) indoor transferring, (c) dressing, (d) toileting, (e) eating, and (f) continence [37]. Response categories for ADL were consistent with IADLs and coded similarly.

We used the previously validated Chinese version of the Mini-mental Status Examination (MMSE) [37] that was modified from the Folstein scale [49] to measure cogitive impairment. Consistent with the original version, the Chinese MMSE included seven following domains: orientation, short-term memory, reaction, calculation, drawing, naming, and language. It has a total score of 30. A respondent was classified as cognitively impaired if he/she had an MMSE score less than 24 (coded as 1); otherwise, he/she was considered cognitively unimpaired [49]. As the level of educational attainment among Chinese older adults is relatively low, an alternative criterion score of 18 was used to assess sensitivity. The results were largely similar.

##### Mortality risk

All-cause mortality risk was defined by the survival status (alive or dead) at the time of the 2014 survey and the length of exposure to mortality. The length of exposure was measured by the number of days survived. For those who died during the study period, exposure was counted from the date at the initial interview (in 2005–2014) to the date of death. For respondents who were alive in 2014, the exposure was counted from the initial interview to the date of the 2014 survey. The date of death was ascertained from the official certificates of death (when available); otherwise, it was obtained from either next-of-kin or informant and was confirmed by a local residential committee. From 2005 to 2014, approximately 50% of individuals died before the 2014 survey (27% in the weighted data) and about 21% of study participants were lost during follow-up (28% in the weighted data). The quality of mortality data has been shown to be high in the CLHLS [37].

#### Covariates

All analyses were stratified by sex and age group (65–74, 75–84, 85–94, and 95+). The analyses also adjusted for a number of demographic, socioeconomic, and behavioral factors that have been shown to be associated with access to care [31, 35, 50–53], disability, cognitive impairment, and overall mortality [2, 17, 54, 55]. Further detail of the coding of study covariates have been documented extensively elsewhere online [14].

#### Analytical strategy

To investigate the concurrent associations between access to care and the three major health outcomes—IADL disability, ADL disability, and cognitive impairment, we used mixed-effect logistic regression models. This approach is a variant of the classic multilevel analysis—with unbalanced data and observations at the first level and individuals at the second level—and is common in social science research and aging studies [56–60]. Random effect (random intercept) models were applied. We used nested models to assess how different sets of covariates influenced the associations. Model I included sociodemographic background, year of the interview year, and proxy responses to access to healthcare. Model II further added whether a respondent was enrolled in the national health insurance program. Participants’ health behaviors were further added in Model III.

We used multivariate hazard regression models to investigate the longitudinal relationship between access to care and the subsequent risks of mortality in the period of 2005–2014. A Weibull hazard function was chosen because preliminary analyses indicated it provided the best overall model fit (vs. exponential, Gompertz, lognormal, etc.) based on BIC values; and because some variables violated Cox’s proportionality assumption. Respondents who only contributed one interview (in either the 2005, 2008 or 2011 wave) and were subsequently lost to follow-up were excluded from the analysis. For respondents who contributed two or more interviews and were subsequently lost to follow-up afterwards, their exposure to mortality during the interval before their last interview was treated as censored. An alternative approach also used multiple imputation for those who contributed one interview yet were lost to follow-up and assumed that these individuals had the same survival status (and length of exposure) as those with known survival status—with the same demographics, psychosocial characteristics, and health conditions. The analyses yielded similar results to the un-imputed models (results available upon request).

The final analytic sample for the hazard models included 19,785 individuals with valid information on their survival status and the duration of exposure to mortality risk. Several nested models were used in an approach consistent with the analyses for IADL, ADL, and cognitive impairment. For the mortality outcome, Model IV also was added to adjust for health status at baseline. Due to space constraints, the estimated ORs and HRs for the individual covariates were not presented in the tables but are available upon request.

Inadequate access to care and all covariates in the analytic models of IADL, ADL, and cognitive impairment were incorporated (whenever possible) as time-varying indicators; whereas these variables were fixed at baseline when examining the prospective risks of mortality. Data among study variables was missing in less than 2% of cases and listwise deletion was used in all models. Alternative approaches to imputation were evaluated (e.g., multiple imputation, mean/modal imputation, etc.) and the findings were consistent (results are available upon request). Multicollinearity among covariates also was assessed in preliminary analyses and no issues were detected [61].

The analyses were stratified by age group and sex to count for the well-documented differences in sociodemographic background, health status, and access to care described above. Preliminary analyses of the health outcomes also indicated statistically significant interactions between access to care and various age-sex groups to justify the stratification approach. The -suest- command in Stata was used to assess significant differences in the associations between inadequate access to care and health outcomes across age groups in men and women and by sex in the age groups. We applied the sampling weights to all models to account for the CLHLS study design. We relied on the statistical software of Stata version 15.0 to fulfill our research goals.

## Results

Table 1 presents the weighted percentages of the study sample and by age and sex groups. Although the majority of older adults reported adequate access to care, approximately 6.5% of respondents reported that they lacked adequate access to care services during the period 2005–2014. With the exception of young-old adults (aged 65–74), women were generally more likely to report inadequate access to care than men. The percentage of those reporting inadequate access to care also exhibited an increase across advancing age groups. As expected, overall levels of disability, cognitive impairment, and mortality increased across age groups in men and women. Table 1 also shows that adults at older ages were more likely to live with their children and were less likely to have a spouse, had lower SES, were less likely to smoke, and were less likely to engage in various leisure activities. The age patterning of the distributions was largely similar in men and women; however, women exhibited generally greater levels of disadvantage relative to men with regard to socioeconomic standing and health status.

**Table 1:** Weighted Percentages of Study Variables by Sex and Age Group among Older Adults in China, CLHLS, 2005–2014

		Women				Men			
	Total	Ages 65–74	Ages 75–84	Ages 85–94	Ages 95+	Ages 65–74	Ages 75–84	Ages 85–94	Ages 95+
#, Total observations	46,549	4137	5264	7718	9181	4633	5548	6772	3296
#, Total participants	25,567	2149	2206	4439	6023	2463	2409	3910	1968
Access to Healthcare									
% Self-report Inadequate access to healthcare	6.5	5.7	7.3	9.2	9.2	6.6	6.1	7.6	8.0
Health Outcomes									
% IADL disabled	36.3	30.2	59.9	85.1	94.8	18.3	39.6	70.1	90.0
% ADL disabled	10.0	6.4	13.0	26.4	47.4	7.3	12.2	21.4	42.8
% Cognitive impaired	14.0	9.9	24.0	46.7	70.3	6.5	14.0	29.7	54.1
% Died in the period 2005–2014	26.8	15.1	37.9	62.6	80.3	21.0	44.0	67.5	80.3
Sociodemographic Background									
Mean Age (in years)	73.5	69.4	78.7	87.8	97.0	69.3	78.5	87.5	96.9
% Men	47.9	–	–	–	–	–	–	–	–
% Urban	44.6	44.8	43.6	43.7	46.7	44.0	45.4	47.4	52.4
% Currently married	63.3	64.9	35.5	14.3	3.5	82.6	69.9	44.4	21.5
% Coresidence with children	43.7	44.9	51.0	65.8	77.4	37.5	38.6	49.2	67.7
% 0 years of schooling	42.0	48.6	73.3	84.4	88.1	28.5	29.4	39.3	44.8
% 1–6 years of schooling	40.4	39.2	21.5	12.7	9.7	50.5	50.6	46.4	42.7
% 7+ years of schooling	17.6	12.3	5.2	2.9	2.2	31.2	20.0	14.3	12.4
% White collar occupation	11.6	7.5	7.9	5.5	5.1	16.0	16.8	12.6	12.5
% Economic independence	46.2	45.8	24.4	12.3	6.8	64.7	46.4	31.5	29.3
National Health Insurance									
% Enrolled in NCMS	51.9	49.8	55.9	58.1	55.8	50.7	51.8	53.4	51.5
% Enrolled in UMS	21.6	20.7	16.8	12.8	12.7	23.8	26.4	22.7	24.7
Health Behaviors									
% Currently smoking	24.5	7.4	6.9	5.9	5.6	47.2	37.9	31.7	22.6
Leisure activities (range = 0–24)	11.9	12.7	10.5	7.4	4.5	12.9	11.4	8.8	5.9
Survey Measures									
% Wave 2005	20.8	22.8	18.3	14.7	12.3	23.1	17.7	13.0	9.1
% Wave 2008/2009	25.5	25.3	24.7	24.6	25.2	26.7	24.5	24.0	26.5
% Wave 2011/2012	29.2	28.0	30.2	31.8	34.3	28.2	31.2	33.6	39.2
% Wave 2014	24.6	23.9	27.0	28.9	28.3	22.0	26.6	29.5	25.2
% Proxy response for access to healthcare	5.1	2.7	7.3	21.4	43.0	2.7	5.9	15.3	36.3

Abbreviations: *IADL* instrumental activities of daily living, *ADL* activities of daily living, *NCMS* New Cooperative Medical Scheme, *UMS* urban medical schemes

Note: The weighted percentages were based on the number of observations and were very similar to percentages based on the number of individuals—with the exception of the distributions for survey wave, percentage of death, and enrollment in national health insurance. The numbers of total observations and participants were unweighted

Odds ratios (ORs) are presented in Table 2 for IADL, ADL, and cognitive impairments associated with reported inadequate access to care by age and sex groups. Overall, the findings suggest that those who reported inadequate access to care had significantly greater odds of IADL disability (OR = 2.05, 95% CIs: 1.76–2.39), ADL disability (OR = 2.47, 95% CIs: 1.83–3.35), and cognitive impairment (OR = 2.49, 95% CIs: 2.15–2.96) compared to those who had adequate access to care when sociodemographic factors were adjusted for (Model I). The ORs were only partially reduced and remained significant for IADL disability (OR = 1.79, 95% CIs: 1.51–2.12), ADL disability (OR = 1.71, 95% CIs: 1.30–2.26), and cognitive impairment (OR = 1.93, 95% CIs: 1.63–2.27) after adjusting for sociodemographic background, health insurance, and health behaviors in Model III. Table 2 also shows that the association between reported access to care and the health outcomes varied for men and women and across age.

**Table 2:** Odds Ratios (95% Confidence Intervals) of IADL Disability, ADL Disability, and Cognitive Impairment for Self-report Inadequate Access versus Adequate Access to Healthcare by Sex and Age Group among Older Adults in China, CLHLS 2005-2014

	Model I	Model II	Model III
IADL Disability			
Total	2.05 (1.76-2.39)***	2.02 (1.73-2.37)***	1.79 (1.51-2.12)***
Women, All ages 65+	2.31 (1.88-2.85)***	2.27 (1.83-2.83)***	1.96 (1.55-2.50)***
Women, Ages 65-74	2.29 (1.69-3.11)***	2.22 (1.61-3.06)***	1.95 (1.36-2.80)***
Women, Ages 75-84	2.46 (1.89-3.20)***	2.49 (1.91-3.24)***	2.08 (1.58-2.73)***
Women, Ages 85-94	1.68 (1.13-2.51)**	1.63 (1.10-2.42)*	1.35 (0.91-2.01)
Women, Ages 95+	4.27 (1.99-9.19)***	3.98 (1.85-8.56)***	3.24 (1.50-6.97)**
Men, All ages 65+	1.79 (1.42-2.25)***	1.77 (1.41-2.24)***	1.58 (1.24-2.02)***
Men, Ages 65-74	1.47 (1.02-2.11)*	1.42 (0.98-2.05)+	1.37 (0.94-2.01)+
Men, Ages 75-84	2.42 (1.85-3.16)***	2.50 (1.91-3.27)***	1.96 (1.47-2.63)***
Men, Ages 85-94	2.04 (1.46-2.86)***	2.09 (1.49-2.94)***	1.74 (1.21-2.49)**
Men, Ages 95+	1.75 (0.81-3.80)	1.50 (0.69-3.24)	0.86 (0.35-2.14)
ADL Disability			
Total	2.47 (1.83-3.35)***	2.29 (1.73-3.04)***	1.71(1.30-2.26)***
Women, All ages 65+	3.25 (2.11-5.01)***	2.99 (2.02-4.45)***	2.10 (1.49-2.95)***
Women, Ages 65-74	5.35 (2.28-12.5)***	4.78 (2.25-10.1)***	3.23 (1.75-5.94)***
Women, Ages 75-84	2.33 (1.68-3.23)***	2.19 (1.59-3.02)***	1.45 (1.05-2.01)*
Women, Ages 85-94	1.94 (1.50-2.51)***	1.86 (1.45-2.39)***	1.47 (1.14-1.90)**
Women, Ages 95+	1.77 (1.33-2.34)***	1.68 (1.28-2.21)***	1.35 (1.02-1.77)*
Men, All ages 65+	1.68 (1.13-2.50)*	1.59 (1.10-2.29)*	1.25(0.86-1.81)
Men, Ages 65-74	1.01 (0.42-2.43)	0.99 (0.47-2.10)	0.87 (0.40-1.91)
Men, Ages 75-84	2.56 (1.83-3.59)***	2.60 (1.86-3.63)***	1.77 (1.27-2.46)**
Men, Ages 85-94	2.37 (1.72-3.26)***	2.31(1.68-3.19)***	1.72 (1.24-2.41)**
Men, Ages 95+	1.76 (1.16-2.67)**	1.67 (1.10-2.54)*	1.14 (0.75-1.70)
Cognitive Impairment			
Total	2.49 (2.12-2.92)***	2.34 (1.99-2.74)***	1.93 (1.63-2.27)***
Women, All ages 65+	2.64 (2.14-3.27)***	2.47 (2.00-3.05)***	2.04 (1.64-2.53)***
Women, Ages 65-74	2.94 (1.95-4.45)***	2.59 (1.73-3.89)***	2.10 (1.38-3.19)**
Women, Ages 75-84	2.66 (2.07-3.41)***	2.59 (2.02-3.31)***	2.13 (1.65-2.76)***
Women, Ages 85-94	1.66 (1.30-2.12)***	1.59 (1.24-2.04)***	1.35 (1.03-1.75)*
Women, Ages 95+	1.35 (0.96-1.91)+	1.27 (0.90-1.79)	1.02 (0.70-1.49)
Men, All ages 65+	2.23 (1.72-2.90)***	2.12 (1.64-2.74)***	1.74 (1.35-2.25)***
Men, Ages 65-74	1.91 (1.18-3.09)**	1.84 (1.15-2.95)*	1.73 (1.10-2.75)*
Men, Ages 75-84	2.73 (2.05-3.63)***	2.57 (1.93-3.43)***	1.91 (1.42-2.57)***
Men, Ages 85-94	2.28 (1.70-3.05)***	2.18 (1.63-2.91)***	1.75 (1.29-2.37)***
Men, Ages 95+	2.37 (1.49-3.79)***	2.21 (1.38-3.56)**	1.63 (1.02-2.61)*

Note: Estimated odds ratios (ORs) were weighted and adjusted for within-person correlation. The total analytic sample included 48,476 observations from 26,604 individuals. Model I controlled for sociodemographic factors. Model II added two measures for enrollment in national health insurance programs. Model III further included measures for health behaviors. All models controlled for survey year and proxy responses to the question of adequate access to healthcare. Due to space constraints, the ORs for covariates are not reported for the 99 estimated models (available upon request)

+*p*<0.1, *p<0.05, **p<0.01, ***p<0.001

In terms of sex differences, we found that the odds of IADL disability for reported inadequate access to care were slightly higher in women (ORs = 1.96–2.31) than in men (ORs = 1.58–1.79) across Models I-III (*p* < 0.10 for difference). Similarly, the odds of ADL disability were moderately higher in women (ORs = 2.10–3.25) than in men (ORs = 1.25–1.68) across models (*p* < 0.05 for difference). However, we found no significant difference between men and women in the association between cognitive impairment and inadequate access to care.

In terms of age differences, the ORs for IADL disability were largest at the oldest-old ages (ages 95+) in women (ORs = 3.24–4.27) and at middle-old (ages 75–84) or old-old (ages 85–94) age groups in men (ORs = 1.74–2.42). We found no association between IADL disability and reported access to care in men at the oldest-old ages. For ADL disability, the ORs in women were more pronounced at young-old ages (ages 65–74) and middle-old ages (75–84) compared with women at other ages (*p* < 0.05 for difference). In contrast, the ORs for ADL disability in men were most pronounced at middle-old ages and old-old ages compared with men at other ages (*p* < 0.05 for difference). With regard to cognitive impairment, results indicate age differences in the associations for women but not for men. The ORs for cognitive impairment in women were largest at young-old and middle-old ages compared with women at old-old and oldest-old ages (*p* < 0.01 for difference).

Table 3 presents hazard ratios (HR) for the associations between reported inadequate access to care and prospective mortality by age and sex groups. Results show that older adults in China with inadequate access to care had significantly greater mortality risks (HRs = 1.22–1.33) than those with adequate access to care after adjusting for sociodemographics, health insurance, and health behaviors. The association was further attenuated after adjusting for health status at baseline in Model IV (HR = 1.17, 95% CIs: 1.04–1.32). The mortality risks were similar in men and women; however, we found that the increased HRs were primarily among women ages 65–74 and 75–84 and primarily among men ages 75–84 and 85–94—which is similar to the age patterning we found for ADL disability.

**Table 3:** Relative Hazard Ratios (95% Confidence Intervals) of Mortality for Self-report Inadequate Access versus Adequate Access to Healthcare by Sex and Age Group among Older Adults in China, CLHLS 2005-2014

	Model I	Model II	Model III	Model IV
Total	1.33 (1.17-1.49)***	1.30 (1.15-1.47)***	1.22(1.08-1.38)**	1.17 (1.04-1.32)*
Women, Ages 65+	1.32 (1.12-1.56)**	1.30 (1.10-1.54)**	1.21 (1.03-1.44)*	1.14 (0.96-1.35)
Women, Ages 65-74	1.42 (1.00-2.05)*	1.38 (0.97-1.95)+	1.24 (0.87-1.78)	1.16 (0.81-1.67)
Women, Ages 75-84	1.38 (1.09-1.74)**	1.36 (1.08-1.72)**	1.28 (1.01-1.62)*	1.18 (0.93-1.50)
Women, Ages 85-94	0.99 (0.83-1.18)	0.99 (0.83-1.18)	0.92 (0.77-1.11)	0.89 (0.74-1.08)
Women, Ages 95+	1.14 (0.95-1.36)	1.13 (0.95-1.35)	1.07 (0.90-1.29)	1.04 (0.86-1.24)
Men, Ages 65+	1.30 (1.09-1.55)**	1.29 (1.08-1.54)**	1.22 (1.02-1.45)*	1.20 (1.00-1.43)*
Men, Ages 65-74	1.29 (0.97-1.72)+	1.28 (0.96-1.71)	1.21 (0.91-1.62)	1.18 (0.88-1.57)
Men, Ages 75-84	1.36 (1.08-1.72)**	1.33 (1.04-1.67)*	1.22 (0.96-1.54)+	1.21 (0.96-1.51)
Men, Ages 85-94	1.22 (1.01-1.46)*	1.22 (1.02-1.47)*	1.18 (0.99-1.42)+	1.19 (0.99-1.43)+
Men, Ages 95+	1.16 (0.88-1.52)	1.13 (0.87-1.48)	1.14 (0891-1.45)	1.18 (0.92-1.50)

Note: Relative hazard (RHs) ratios were weighted and estimated from Weibull regression models. The total analytic sample included 20,532 individuals (*n*=11,830 for women; *n*=8,702 for men). Model I controlled for sociodemographic background. Model II added two measures for enrollment in national health insurance programs. Model III added measures for health behaviors and Model IV further added measures for baseline health status (IADL disability, ADL disability, and cognitive impairment). All models controlled for survey year and proxy responses to the question of adequate access to healthcare. All covariates were fixed at the first interview in the period 2005-2014. Due to space constraints, the HRs for covariates are not reported for the 44 estimated models (available upon request). +*p*<0.1, *p<0.05, **p<0.01, ***p<0.001

## Discussion

Using a uniquely large nationally representative sample of adults aged 65 and older in mainland China, our study investigated the contemporaneous associations between reported access to medical care and physical and cognitive functions in terms of ADL disability, IADL disability, and cognitive impairment. The prospective relationship between access to care and subsequent mortality from 2005 to 2014 was also examined. To our knowledge, this study is the first systematic investigation of how inadequate access to care is related to physical functions, cognitive function, and mortality in a robust longitudinal sample of adults aged 65–74 (*n* = 4612), 75–84 (*n* = 4615), 85–94 (*n* = 8349) and 95+ (*n* = 7991). Overall, we found that relative to those who reported adequate access to care, older adults who reported inadequate access to care had a substantially higher risk of IADL and ADL disabilities, cognitive dysfunction, and subsequent mortality. The associations were generally stronger in women than in men, varied across age groups, and largely persisted despite taking into account multiple sociodemographic factors, behavioral factors, and health-related factors.

Previous studies have shown that self-reported access to care is critical for maintaining the health status of older adults. Self-assessed reports of adequate access to care reflect the perceived and actual availability of timely care to slow the progression of disease, maintain immune function, and ultimately prolong survival [44–47]. Building on this line of research, we found that older adults in China who reported a lack of adequate access to care were significantly more likely to exhibit physical disability, cognitive impairment, and death relative to older adults who did not report inadequate access to care. The overall associations are robust and are generally compatible with prior studies showing a relationship between health insurance coverage [4, 5, 7, 55, 62] or utilization [63, 64] and various indicators of physical health and mortality. Moreover, we showed that the association between reported (in)adequate access to care and health outcomes was independent of whether the individual had health insurance coverage. This suggests that measures of health insurance status alone may be insufficient for understanding health disparities among adults at older ages. Due to the unavailability of data on healthcare utilization in the CLHLS, we were not able to determine whether the association was also independent from actual healthcare use in the current study. We encourage more research on this topic to better understand the interconnectedness among self-reported access to care, actual use, and subsequent health outcomes.

Our findings also suggested that self-reported inadequate access to care had somewhat greater consequences for the physical, cognitive, and overall survival status of older women in China than older men. Possible explanations for the sex differences are twofold. First, older women generally have less education and fewer economic resources than older men [31, 65]. This is especially the case for older women in China who were exposed to sexism at younger ages due to cultural norms and traditional social system [66]. Women also generally have higher levels of disability and greater overall health needs than men [28, 30]. Together, the greater need for care in the absence of resources may lead to delays in care—and/or lower-quality care—that may exacerbate health decline in women [12, 53, 67], which in turn, produces wider health disparities in women attributable to inadequate access to care. Second, although women are more likely to use preventive care, outpatient care, and community/home-based services than men [19, 28–30, 32, 34, 68], some studies suggest that women generally receive less inpatient care and less aggressive treatments than men [33, 69, 70]. On one hand, women with adequate access to preventive, outpatient, and community/home care will have conditions diagnosed and treated at younger ages. On the other hand, inadequate access to inpatient care and/or aggressive treatments may exacerbate poorer health outcomes at older ages in women relative to men [71, 72]. We encourage additional studies to explore these factors and other possible explanations for the sex differences found in this study.

Another key finding was that reported access to care had a stronger association with health outcomes among women at ages 65–84 and among men at ages 75–94 compared with their respective age-sex counterparts. Although there is limited evidence to support these findings, several explanations are posited. First, the oldest-old adults in this study are characterized by the lowest levels of health and greatest vulnerability to sickness and death relative to younger older adults. Therefore, it can be argued that the distinction between reporting adequate versus inadequate access to care may not produce declines as pronounced as in younger ages in disability, cognitive impairment, and/or mortality at these advanced ages. Second, differences in evidence-based practice and treatment regimens may potentially influence age-patterning of health outcomes related to care in older men and women [73, 74]. Studies have shown that women ages 65–84 may be prescribed more medications than women at oldest-old ages [75]. Likewise, other studies have shown that very old adults are more likely to receive inappropriate medication prescriptions compared with older adults at younger ages [76]. Third, there may be important unmeasured physiological and/or genetic components at play that warrant additional investigations to explain the observed differences among men and women at differing ages [30, 41, 77, 78]. Finally, it is also possible that young- and middle-old women and middle- and old-old men may have distinct perceptions about what it means to have (in)adequate access to care; which in turn, may be related to their health status. To be sure, we strongly encourage future studies to further validate these findings and explore these and other possible mechanisms contributing the associations.

It is worth noting that while the relationship between inadequate access to medical care and mortality was longitudinal in this study, the relationships between inadequate access to care and IADL/ADL disabilities and impaired cognition were contemporaneous. In doing so, we focused on the prevalence of physical disabilities and cognitive impairment rather than the incidence (or recovery) from these statuses. A major reason for our current focus is that (in)adequate access to care is highly endogenous with the incidence (and recovery) of IADL and ADL disabilities and cognitive impairment. Inadequate access to care may contribute to poorer health; however, it is also possible that people in poorer health are likely use more healthcare—a somewhat counterintuitive phenomenon noted in the literature [7, 43]. Thus, the greater use of healthcare may result in a high rate of reportedly adequate access to care. Another reason is that the observation period is relatively short (about 2–3 years) and that not all episodes of IADL and ADL disabilities and/or changes in cognitive function were gathered in the CLHLS. These limitations prohibited us from appropriately modeling the potential longitudinal relationship between access to care and subsequent health status. By contrast, focusing on the contemporaneous associations better ensured that our results were more conservative. In doing so, the prevalence of IADL/ADL and cognitive impairment may indeed reflect the total (cumulative) association over the entire observation period—which may better capture the long-term effects of having access to health.

A notable strength of our analysis is the use of the largest national survey of adults aged 65 and older from China to examine health outcomes over almost a 10-year period (2005–2014). By using an established high-quality sample [37] that included more than 25,500 individuals with more than 46,500 observations in 2005–2014, we were able to produce robust estimates by age and sex groups at very old ages. Another unique contribution of the study is the application of a person-centered measure of access to care in examining the relationship between access to care and multiple health outcomes. This practice is a departure from prior research that has focused primarily on either having medical insurance or on the actual utilization of healthcare. Research has suggested that it is difficult to make comparisons of health outcomes between insured and uninsured adults because the association between having insurance and health outcomes is complex. On one hand, healthy people may not buy health insurance because of the perception of their own health and needs. On the other hand, individuals in poor health may be forced to buy health insurance to receive needed care [54]. Other evidence has further revealed that actual utilization may confound the role of healthcare or medical care (as an endogenous factor) between utilization of health care and health outcomes [43]—i.e., that people with complex comorbidities exhibit higher patterns of utilization [79, 80]. These complexities may partially contribute to the phenomenon that many studies in different populations find no significant benefit for having health insurance in reducing mortality [81–84]; and in some instances, access to care is counterintuitively related to poor or worsening health status [43]. The (self-reported) measure of access to medical care used in the present analysis minimized issues of endogeneity and captured additional information related to accessing care that a measure of utilization does not capture [44–47].

Another strength of the present study was to investigate several health outcomes related to access to care using multiple (pooled) waves of longitudinal data. This multiwave analytical approach facilitates the examination of the concurrent associations over time and accounts for possible individual time-varying characteristics to better model the associations and confounding effects. We know of only one study so far that has investigated the relationships between self-reported access to care and health outcomes among older adults in urban and rural China [14]. Building upon this research, we further demonstrated strong links between reported access to medical care and risks for functional disability, cognitive dysfunction, and subsequent mortality. Furthermore, we have shown that the major findings were not uniform for men or women, varied with age, and remained largely significant even taking a wide array of covariates into consideration.

The findings from this study have potential implications for improving access to medical care in contemporary China. Although the state-sponsored health insurance schemes have made substantial advances in terms of coverage [85], there have been challenges in access healthcare services in both urban and rural areas because of population aging, skyrocketing costs of prescription drugs and healthcare services, shortage of trained healthcare providers, reduced caregiving resources from family [14]. According to the CLHLS, about 60% of the older adults in China who did not seek outpatient services reported the medical cost as the main reason, although the proportion was reduced to 50% in 2011. How to address these challenges is a key to maximize the beneficial effects of adequate access to care on health. Based on the findings from the present study, older adults seem to face disproportionate challenges in obtaining adequate healthcare with advancing age—particularly in women—relative to older adults at comparatively younger ages. Moreover, our results suggest that reported inadequate access to care is particularly harmful to health at certain ages and may be especially detrimental to women. It may be that increasing benefits from the unification of health care programs may be viable strategies to help reduce the health disparities among age- and sex-groups of older adults in China.

There are some limitations should be taken into account when interpreting our findings. First, we used a self-reported indicator for (in)adequate access to medical care, which is less likely capture the actual utilization of medical services and may be related to the participants’ demographics, socioeconomic status, health literacy, health status, and prior experiences with the healthcare system. Nevertheless, evidence shows that one’s self-assessment of access to care is highly correlated with an individual’s actual utilization of mental health services, routine checkups, or emergency care [8, 86, 87]. Nevertheless, we acknowledge that a self-reported measure of access to care is more directly related to being able to obtain one’s needed medical services—rather than the actual utilization of care [12]. However, self-reports of access to care have the advantage of capturing “real-world” dimensions of accessing care as they relate to possible barriers, physician-patient relations, quality of services, unmet needs, and so forth [44–47]. Relative to this, is how to address differences in reporting patterns. For example, some researchers have used “anchoring vignettes” to adjust for reporting differences in awareness and attitudinal outcomes [88]. However, this approach has not been widely adopted in health services research and the CLHLS did not collect such data—therefore, we were unable to directly address the potential confounding effects of reporting bias. We call for additional studies with similar (or alternative) indicators of self-reported access to care (e.g., vignette approach) to validate the recent findings.

Second, we did not distinguish possible gradients of self-reported access to care due to the unavailability of data. In other words, reported access to care likely falls on a continuum on the level of access; thus, the implications of various levels of access may be different. Future studies should investigate to what extent different degree of one’s access to medical care is related to specific health outcomes. Third, we could not incorporate the severity of each health outcome in our analysis. Because individuals in a poorer health condition generally require more medical care, the associations between access to care and health conditions may have been more robust if the severities of conditions were taken into account. Finally, lack of data prohibited us from further taking into account contextual factors, barriers to healthcare (such as lack of transportation, long distance, high cost of use, etc.), availability of services, and quality of care [51], some of which may be linked to mortality and comorbidity [36]. Further research is clearly warranted to examine such factors to better elucidate the linkages between access to medical care and health outcomes by age and sex among older adults.

## Conclusions

Inadequate access to medical care services is related to significantly higher risks of functional disability, cognitive dysfunction, and subsequent mortality among older adults in mainland China. The relationships were generally stronger in women than in men, and generally stronger among women ages 65–84 and among men ages 75–94 than in their respective age-sex counterparts. These findings underscore the significance of achieving healthy aging by providing adequate medical care services to older adults—especially to those prior to advanced ages. In the context of near-universal healthcare coverage in China, coupled with rapid population aging, our findings suggest areas for further improvement in access to care for men and women at vulnerable ages for achieving healthy longevity.

## Abbreviations

ADL: Activities of daily living
CLHLS: Chinese longitudinal healthy longevity survey
HR: Hazard ratio
IADL: Instrumental activities of daily living
MMSE: Mini-mental status examination
NCMS: New cooperative medical scheme
OR: Odds ratio
SES: Socioeconomic status
UEMS: Urban employer-sponsored medical scheme
UMS: Urban Medical Scheme
URMS: Urban Resident Medical Scheme
